# Ye Are All Physicians of No Value

**Published:** 1853-02

**Authors:** 


"*Ye are all Physicians of no value.*"—The editor of the Philadelphia Med. and Surg. Journal states that "Among the sermons delivered under the auspices of the Medico-Chirurgical College, on Sunday evenings, to medical students, was one by the Rev. W. Bacon Stevens, M. D., on the above text." The editor adds, "It was one of the most ingenious, learned, and eloquent of the season."

We trust the text has no special significance as applied to the physicians of the city in which the sermon was delivered! Possibly the preacher limited its application to irregular practitioners. The auditory must have been taken aback somewhat at the information contained in the announcement of the text.

